# Opportunity Costs Pacifism

**DOI:** 10.1007/s10982-020-09390-7

**Published:** 2020-05-26

**Authors:** James Pattison

**Affiliations:** Department of Politics, School of Social Sciences, University of Manchester, 4.052, Arthur Lewis Building, Oxford Road, Manchester, M13 9PL UK

## Abstract

If the resources used to wage wars could be spent elsewhere and save more lives, does this mean that wars are unjustified? This article considers this question, which has been largely overlooked by Just War Theorists and pacifists. It focuses on whether the opportunity costs of war lead to a form of pacifism, which it calls ‘Opportunity Costs Pacifism’. The article argues that Opportunity Costs Pacifism is, at the more ideal level, compelling. It suggests that the only plausible response to Opportunity Costs Pacifism applies in highly nonideal circumstances. This has major implications for Just War Theory and pacifism since it is only at the highly nonideal level that war can be justified.

## Introduction

In addition to the destruction and devastation caused by the War on Terror and the wars in Iraq and Afghanistan, they have been estimated to have cost the U.S. $5.9 trillion.^1^ This money could conceivably have been used instead to reduce significantly the deaths from preventable diseases globally, potentially saving millions of lives. Does this mean that these wars were wrong, independent of the other reasons for and against them? More generally, if the resources used to wage war could be spent elsewhere and save more lives, does this mean that war is impermissible?

In large part, this issue has been ignored by pacifists and Just War Theorists. Pacifists focus on the critique of war and, in doing so, largely overlook that the resources spent on war could potentially be put to better use.^2^ Similarly, Just War Theorists, for the most part, do not consider opportunity costs. Of those that do consider them, some have claimed that they are outside the domain of Just War Theory.^3^ A handful of Just War Theorists have recently repudiated these claims, persuasively arguing that Just War Theory should consider opportunity costs.^4^ As I discuss below, these accounts, however, tend to underplay the significance of the objection to war that arises from considering opportunity costs. Crucially, they also do not tackle head-on the central issue: do opportunity costs render war impermissible and so lead us to pacifism?

In what follows, I consider whether they do. I explore what I call ‘Opportunity Costs Pacifism’, which holds that opportunity costs render all wars impermissible. I first outline the objection to war provided by opportunity costs and how it relates both to the other central criticisms of war presented by pacifism and to Opportunity Costs Pacifism. I then consider the accounts of opportunity costs offered in Just War Theory, before turning to two responses to Opportunity Costs Pacifism, which I argue fail. I go on to argue that there is a more plausible response to Opportunity Costs Pacifism, but that this applies only in highly nonideal circumstances. This has, I conclude, major implications for Just War Theory and pacifism: it is only at the highly nonideal level that war can be potentially permissible.

Two clarifications are necessary. First, Opportunity Costs Pacifism is concerned with *war*, defined as violent armed conflict involving at least 1,000 battlefield deaths.^5^ It does not *necessarily* hold that the use of force short of war is impermissible. Several of the arguments in favour of Opportunity Costs Pacifism may also apply to the use of force short of war, but making the case against the use of force short of war is beyond the scope of this article, given the variety of measures that this encompasses (e.g. drone strikes, assassinations, and no-fly zones).

Second, for reasons of space, I focus on international threats rather than purely domestic ones. By ‘international threats’, I mean threats posed to the state by another entity from beyond its borders or threats posed to others beyond its borders. This means that Opportunity Costs Pacifism is focused on claiming that international wars are wrong. It does not claim that civil wars are impermissible. Again, it might be extended to this, but this is also beyond the scope of this article, given that civil wars raise additional ethical issues to international wars, such about the rights of non-state actors to wage war and the tactics guerrillas may permissibly adopt.^6^

## Opportunity Costs Pacifism

To start with, it helps to distinguish between the *objection* to war from opportunity costs – what we can call the ‘Opportunity Costs Objection’ – and *the form of pacifism* – Opportunity Costs Pacifism. The Opportunity Costs Objection holds, in short, that war is highly problematic because the resources spent on it could be better spent elsewhere, such as by tackling other crises. Opportunity Costs Pacifism holds that the Opportunity Costs Objection is so serious that it takes us to pacifism. In this section, I will first explicate the Opportunity Costs Objection before considering how it may lead to Opportunity Costs Pacifism.

### A. The Opportunity Costs Objection

There are two central elements to the Opportunity Costs Objection. The first compares *threats*. This is assessed according to what I call below ‘threat-specific considerations’ – the morally relevant features of the threats. Rather than simply assuming that wars tend to tackle the worst situations, we should, the objection runs, look to the magnitude of the threat at stake, judged in terms of the likely impact on individuals’ basic interests. When we do so, the objection continues, there is a huge difference in the magnitude of the threat between war and the other situations that might be tackled instead. Most significantly, communicable and non-communicable diseases threaten far more individuals than the sorts of threats that war might be thought permissible in response to – i.e. terrorism, mass atrocities, and national and collective self-defence. According to the World Health Organization (WHO), the main cause of death globally is ischaemic heart disease (16.6% of all deaths), followed by stroke (10.2%), chronic obstructive pulmonary disease (5.3%), and lower respiratory infections (5.2%).^7^ Moreover, many of these deaths are caused by easily preventable diseases, such as measles (which can be relatively cheaply vaccinated against) and malaria (which mosquito nets can prevent).^8^ Violence only amounts to 2.6% of the annual global death toll. According to the Opportunity Costs Objection, then, if international actors such as states are to take seriously the need to focus on the threats of the largest magnitudes, prioritising violent conflicts is unlikely to be permissible.

The second element of the Opportunity Costs Objection concerns the *justifiability of the measure compared to other measures*. This is assessed according to what I call below ‘measure-specific considerations’ – the morally relevant features of the measure to be adopted. This is a factor, most clearly, of the effectiveness of the response. The Opportunity Costs Objection here is straightforward: many violent conflicts are intractable and military action will often achieve at best only relatively small improvements compared to other forms of action. For instance, even if war saves thousands of lives, this pales into insignificance compared to the millions of lives that could be saved by health interventions. Indeed, the objection runs, notable health interventions with regard to diarrhoea, malaria, smallpox, and other forms of immunisation have *each* saved more lives than if world peace had been secured, with at least 2.5 million deaths per year for each of these four interventions.^9^ Moreover, the objection continues, states should look to where other actors are *not* acting.^10^ They can expect that many states will focus on the intentional violations of basic human rights, given the visceral reaction to them. There is the opportunity to have greater impact where the threat is still serious but receives relatively far less attention, particularly where the victims are underprivileged. This might range from threats to mental health in developing countries to antimicrobial resistance for diseases such as tuberculosis.^11^

It may seem, though, that it is more important to tackle violence in order to promote stability and order, and therefore to reduce poverty and disease in the long term. Indeed, tackling mass atrocities may sometimes play an important role in addressing other issues, such as gender inequality and global poverty, to the extent that security is necessary for development. However, this does not necessarily undermine the Opportunity Costs Objection. This is because poverty can also lead to conflict and so the best way of preventing violence may often be to tackle non-violent harms.^12^ In addition, communicable and non-communicable diseases kill in many stable states and, as noted above, success in tackling conflicts is extremely difficult and so it may be better to tackle the cases where there is no conflict. Furthermore, even if one still holds that addressing violence should be prioritised, this does not mean that violence *against others* should be the focus. The biggest violent killer is self-harm (793,000 deaths in 2016).^13^ This might mean that far more lives could be saved by investing in global mental health than by any other response to violence. Important here is that there is a sizable global ‘treatment gap’; more than 75% of those identified with serious anxiety, mood, impulse control, or substance use disorders in low and middle-income countries receive no care at all, with the treatment gap for schizophrenia and other psychoses in sub-Saharan Africa exceeding 90%.^14^ Even if one still holds that violence against others should be prioritised, interpersonal violence is a far larger killer than what the WHO calls ‘collective violence and international intervention’ – the sorts of situations that war is typically launched in response to (477,000 deaths compared to 184,000 deaths in 2016).^15^ It follows that, on the Opportunity Costs Objection, there should be much greater investment in domestic disarmament, in improving gender equality and campaigns against domestic violence, and in ensuring proper accountability for interpersonal violence.

To be sure, the Opportunity Costs Objection is not necessarily consequentialist. In what I think is its most plausible form, it includes deontological considerations in the assessment of the measures (i.e. as ‘measure-specific considerations’) and these also point to avoiding war. Most clearly, violent responses to violence will often *do* harm to innocents. This falls foul of the Doctrine of Doing and Allowing in moral philosophy, whereby it is widely held that it is worse to *do* harm than it is to *allow* it.^16^ By contrast, non-violent interventions, particularly in global health, can be highly successful and *do* far less harm. To that extent, the Opportunity Costs Objection concerns *negative* duties not to harm. That is, other threats should be tackled to avoid doing harm.

In addition, and perhaps more obviously, the Opportunity Costs Objection concerns *positive* duties to assist. That is, other threats should be tackled to help save more lives. It is worth noting here that virtually all mainstream accounts of international ethics, including those of renowned non-cosmopolitans, accept that states have positive, humanitarian duties beyond their borders.^17^ Yet, few (if any) states currently fulfil these duties sufficiently. For instance, very few meet the very low target of giving 0.7% of their GDP to foreign aid; some of those that do, such as the UK, direct this aid politically, rather than directing it to those most in need.^18^ It follows that it is unlikely that states could invoke having done enough already to fulfil their humanitarian duties in order to overcome the Opportunity Costs Objection.

Nor does it seem that states could plausibly claim that they have tackled all threats and so avoid the Opportunity Costs Objection. The objection is likely to continue to arise because those beyond the state’s borders are almost always at *some* risk of peril. This is because, for the foreseeable future, there will be risks of varying degrees, which cannot be fully eradicated. This is because, more straightforwardly, it seems likely that the resources that publics are willing to support spending on international matters will continue to be limited and so governments will need to prioritise which goals they pursue. It is also because, more fundamentally, the opportunity costs at stake are best understood not as concerning actual harms, but rather threats with varying probabilities of materialising as harm. Some threats of harm will be highly probable, such as climate change-induced global poverty, but others far less so, but still very serious, such as existential risks to the whole of humanity (e.g. a nuclear annihilation and a super-volcanic eruption). It is not possible for states to tackle fully all risks, so that everyone is *fully* protected. They are always going to be subject to *some* existential risk of harm, however small. To that extent, opportunity costs are ubiquitous.

### B. Opportunity Costs Pacifism

Having outlined the Opportunity Costs Objection, let’s now turn to Opportunity Costs Pacifism. This holds that the Opportunity Costs Objection is so serious that wars are impermissible. The reasoning is this: given how poorly wars are likely to fare once opportunity costs are taken into account, pacifism – and, specifically, Opportunity Costs Pacifism – seems to be the correct view. States should ideally use their resources on non-military measures to tackle the most serious threats. So many die from preventable diseases and in other situations that spending resources on wars that save only a few and *do* harm is frivolous. It is hard, the reasoning goes, to identify a *feasible* war, particularly on a large scale, that could be permissible once opportunity costs have been taken into account. It would most likely have to save hundreds of thousands, if not millions, of lives. There does not currently seem to be a threat of sufficient seriousness that war could potentially address and, even if there were, it is unlikely that war would be sufficiently efficacious. After all, World War II – the standard example used against pacifism – was over 70 years ago and it is very unlikely that there will be a similar threat that could be tackled soon. Thus, the argument runs, it seems that the Opportunity Costs Objection takes us to Opportunity Costs Pacifism.

To understand this claim further, it helps to consider how Opportunity Costs Pacifism relates to pacifism and to other pacifist critiques. Although pacifists share a general opposition to war, they vary in how strong this opposition is. On the one hand, on what we can call ‘*soft* pacifism’, the rejection of war is weaker. On this view, militarism is problematic and war is generally wrong and should be avoided, yet occasional wars can still be permissible.^19^ By contrast, on what we can call ‘*hard* pacifism’, the rejection of war is more robust. There are no exceptions – war is impermissible.

One type of hard pacifism is *absolute* pacifism: all wars are *always* impermissible. Another type of hard pacifism is *contingent* (hard) pacifism: all wars are *currently* impermissible – and will be so for the foreseeable future. On this view, as John Rawls notes, “the possibility of a just war is conceded but not under present circumstances”.^20^ Applying this to opportunity costs, (hard) contingent Opportunity Costs Pacifism holds that it is conceivable that war might be permissible because of the opportunity costs, but this is not reasonably foreseeable in current circumstances or in the near future. It does not claim, though, that war will always be impermissible. There might be circumstances in the past where war was permissible or far into the future where war is permissible (or even in the near future, but not currently reasonably foreseeable). To that extent, it is still somewhat tentative.

This (hard) contingent pacifism is the form of Opportunity Costs Pacifism that I will focus on. This is because, first, it is unclear whether the soft rejection of war is any different to dovish forms of Just War Theory. As such, what is most interesting and significant about pacifism – the outright rejection of war as a policy option – is not present. Second, (hard) *absolute* pacifism seems too vehement. It seems plausible that wars in the past were permissible (e.g. World War II) and it is at least conceivable that, in the distant future, there might be an international system where war is the best option, such as if cheap technology allows for highly precise targeting of only those who are fully culpable, with no collateral damage.

The two central reasons in favour of pacifism highlighted by pacifists concern the (1) instrumentalist and (2) the deontological critique of war.^21^ The instrumentalist claim is that, by killing non-combatants, destroying infrastructure, harming international order, and promoting violence, wars have huge negative consequences.^22^ The benefits that war might bring, such as in terms of saving lives with humanitarian intervention or defending sovereignty, do not outweigh these harms. The deontological critique is that wars *do* harm in that they involve killing soldiers and civilians, and thereby fall foul of the Doctrine of Doing and Allowing in moral philosophy, which is sometimes framed by pacifists in terms of the difference between *killing* and *letting die*.^23^

In addition to these central pacifist arguments, which rely on the critique of war, there is an oft-overlooked, third central reason in favour of pacifism. This is a *positive* reason in favour of the alternatives to war, such as targeted sanctions, diplomatic measures, positive incentives, criminal tribunals, humanitarian assistance, and accepting refugees. These options are often more effective than appreciated and have other reasons in their favour. For instance, certain diplomatic measures (e.g. naming and shaming) can contribute to morally important global norms and other measures, such as economic sanctions and arms embargoes, can distribute costs more fairly.^24^ In short, then, war is wrong because (3) the alternatives are typically better.

However, it might be replied that these three points do not lead to pacifism on their own. For instance, it might be claimed (to be clear, I take no stance) that, when judged in isolation, certain recent wars were all-things-considered permissible, such as the First Gulf War in 1991 and the humanitarian intervention in Kosovo in 1999, to the extent that they brought about good consequences and there were not any clear feasible alternatives. In addition, it seems that the deontological critique of war appears to be sometimes over-egged, since the difference between *doing* and *allowing* harm is not absolute. As such, even if wars *do* some harm, they can still sometimes be permissible.^25^

This is where the Opportunity Costs Objection comes in. It adds a fourth central reason in favour of pacifism that pacifists can draw upon, in addition to the critique of war for (1) instrumental and (2) deontological reasons, and (3) the reasons in favour of the alternative measures to address the threat at hand. It concerns the reasons to address (4) *other threats*. This might be thought to take us to pacifism, even in cases such as the First Gulf War and Kosovo. Some might hold that the Opportunity Costs Objection is, on its own, *sufficient* to lead to pacifism. This would be a pure form of Opportunity Costs Pacifism. Yet it seems more plausible that the Opportunity Costs Objection plays a central role *in tipping the balance* in favour of pacifism, together with the other three central reasons. This is a more mixed form of pacifism, but it can still be viewed as Opportunity Costs Pacifism given the central role played by opportunity costs.

## Opportunity Costs and Just War Theory

Having outlined Opportunity Costs Pacifism, let’s now consider how opportunity costs have been viewed in relation to the ethics of war. As noted at the start, Just War Theorists have largely overlooked opportunity costs, with their predominant focus on the permissibility of responding to one particular threat, but some philosophers have recently highlighted that Just War Theory should take seriously opportunity costs. It is worth considering these claims because, on the one hand, they repudiate some potential replies to the Opportunity Costs Objection. On the other, they appear to underestimate the seriousness of the Opportunity Costs Objection and examining why highlights a further reason in favour of Opportunity Costs Pacifism.

In his brief discussion of the issue, Victor Tadros argues that the possibility of using the war chest to fulfil humanitarian duties

provides a powerful reason to think that it is typically wrong to engage in humanitarian wars. The state should rather spend its resources preventing and curing illness and disease, for example by providing mosquito nets, clean water or access to essential medicines. Doing these things saves a great number of people from death and serious illness, killing almost no one.^26^

For Tadros, humanitarian intervention can be wrongful on an ‘internal’ basis, when others have legitimate claims on the resources used to pursue them, in addition to external reasons why intervention might be wrongful, such as the potential for them to harm civilians.

In a similar vein, Kieran Oberman argues that “since states that wage war could alleviate poverty instead, poverty can render war unjust”.^27^ According to Oberman, this is when, first, war would be disproportionate because of its opportunity costs. Second, Oberman argues that opportunity costs can render war wrongful when there is a better means of achieving the same end – the ‘just ends’ of war (I will consider this further below). In large part, Oberman focuses on the issue of whether poverty can make wars of humanitarian intervention impermissible, but also notes that this reasoning might apply to other causes, such as climate change and wars of self-defence.

One response to this claim is that humanitarian intervention is permissible because it tackles intentional killing, whereas poverty is largely unintentional. However, as Peter Singer helpfully argues, there are not adequate grounds for giving priority to tackling genocide (through war) rather than tackling other poverty-related deaths.^28^ This is because, even if tackling intentional harm is more important than tackling unintentional harm, it is not *so* much more important that it justifies saving far fewer lives. For Singer, “if, given our available resources, we could save the lives of many more victims of poverty than we could save, if instead we attempted to save the lives of victims of genocide, we should save the victims of poverty”.^29^ In his (again) brief account of the issue, Jeff McMahan agrees that intentional violations are not far worse.^30^ McMahan goes on to argue that, rather than overlooking opportunity costs in Just War Theory, there should be, in fact, a new opportunity costs principle of Just War Theory.

Although these accounts are helpful in showing why opportunity costs should be taken seriously by Just War Theory, they do not fully consider whether the opportunity costs of war are so serious that they undercut the possibility of just war and lead to Opportunity Costs Pacifism. (Indeed, as I consider further below, Oberman argues that opportunity costs do *not* take us to pacifism.) One reason why this appears to be the case concerns the ways that opportunity costs are conceived in relation to Just War Theory in these accounts. That is, they appear to underplay the significance of the measures used in response to tackle other threats.

To explicate, we need to distinguish between two types of consideration, which I mentioned briefly above. The first are *threat-specific considerations*. These concern the morally relevant features of the threat. Although space precludes providing a detailed account of these, they concern features that are widely thought as relevant in related fields, such as in how to prioritise limited resources in healthcare.^31^ Leading threat-specific considerations include the size of the threat in terms of the number of individuals affected, the magnitude of the threat to the individuals (e.g. if it concerns their right to life or relatively minor rights), the probability of the threat materialising, and the culpability of those threatened.

The second are *measure-specific considerations.* These concern the morally relevant features of the measures that will be used to address the threat, such as whether a measure will be non-coercive. Again, space precludes a detailed account here of these considerations, but they concern features that are widely seen as relevant in the assessment of measures used to address threats, such as of war and its alternatives. Leading measure-specific considerations include the efficacy of the measure at addressing the situation at hand, the broader consequences of the measure, whether the measure receives the consent of the intended beneficiary, how the measure will distribute costs, whether the measure *does* harm, and if so whether the measure’s harm will be mediated by another’s intervening agency.^32^

Accounts of opportunity costs in the above accounts focus largely on the threat-specific considerations. They do not sufficiently appreciate the measure-specific ones. Both are central. To see this, consider the following scenario.

*Ground Operation*: France could reduce the threat posed by a terrorist group to itself by initiating an extensive ground operation in Burkina Faso. Such an operation would use significant political and economic resources and be likely to harm several innocent civilians. It could, however, use these political and economic resources instead to offer a large financial inducement to the parties involved in mass atrocities in South Sudan. This would incentivise them to stop their killing and lead to a negotiated settlement. Given budgetary and political constraints, it is possible for France to undertake only one measure.

In this example, it is not enough to look to the threat-specific considerations to decide which course of action France should adopt. The threat-specific considerations concern whether a terrorist threat to France from inside Burkina Faso or mass atrocities in South Sudan should be the priority. In addition, France needs to look to the measure-specific considerations. These concern the measures that would be used to address the threats in Burkina Faso and South Sudan. These might appear to point in favour of tackling the situation in South Sudan, given that the inducements would be less harmful, more effective, and less costly than military action.

As such, the measures used – the alternatives to war – are central to assessing opportunity costs. The case for focusing, for instance, on communicable and non-communicable disease is significantly strengthened by the means that will be used to address these threats. As noted above, health interventions are typically far less coercive and more effective than military actions.^33^ This is in addition to the far greater seriousness of the threat posed by communicable and non-communicable disease.

The centrality of the alternatives to war to the assessment of opportunity costs has not hitherto been fully appreciated in the handful of discussions of opportunity costs in Just War Theory. Part of the reason it seems is that it is, on the face of it, unclear how opportunity costs should fit within Just War Theory. They appear to be most clearly associated with the principles of proportionality and necessity, which concern the costs of war and alternative courses of action.^34^ Suppose that one sees them as a matter of proportionality. Opportunity costs could render war as disproportionate because war uses resources that should be spent elsewhere. However, proportionality is widely understood as the case for performing an action *compared to inaction* – i.e. compared to not waging war against the terrorist group in Burkina Faso – rather than how the action *compares to performing other actions – *i.e. to offering incentives to those involved in mass atrocities in South Sudan.^35^ It is unclear how opportunity costs can then be included into proportionality.

Suppose instead that one sees opportunity costs as a matter of necessity. Necessity, as understood in recent accounts of Just War, compares the case for performing an action to other actions that could be used to redress the threat at hand. Necessity is sometimes used as a synonym for last resort, which also requires a comparison of war to other measures. On Seth Lazar’s leading account of necessity, “[w]here an option *O* aims to avert a threat *T*, we determine *O*’s necessity by comparing it with all the other options that will either mitigate or avert *T*”.^36^ However, the problem is that necessity, as understood here, concerns the measures that will address *a specific threat*, ‘*T*’, such as mass atrocities. It does not compare measures that will be used to address *other threats*. This is exactly what is called for with opportunity costs. For instance, *Ground Operation* concerns the tackling of different situations – either reducing the terrorist threat facing France or helping to address mass atrocities in South Sudan. The decision facing France is not choosing the best means of addressing *one specific threat*, but rather choosing the best response to *multiple threats* to different sets of individuals – those under terrorist threat in France compared to those facing mass atrocities in South Sudan. This choice cannot therefore be easily captured by the principle of necessity, at least as it is commonly understood.

Given the problems of relying on necessity or proportionality, we could instead look to a new principle of opportunity costs, as McMahan proposes.^37^ This would concern which threat should be addressed. In *Ground Operation*, it would consider which situation is the most pressing to tackle: the threat from the terrorist group in Burkina Faso or mass atrocities in South Sudan? This might appear to be the most straightforward option, especially when used prior to the assessment of necessity.^38^ Here is how one might conceive the decision-making. First, it is necessary to determine which is the most serious situation. Suppose that in *Ground Operation* this is the threat posed by the terrorist group in Burkina Faso. Second, it is necessary to determine the best measure to tackle the terrorist group, such as whether there should be economic sanctions, drone strikes, or a ground invasion. Let’s suppose that the best measure is indeed a ground operation in Burkina Faso, which is most likely to be successful and will ultimately cause the least amount of harm to innocent individuals.

The problem, though, is that such sequential decision-making makes a crucial error. It misses comparing measures across multiple threats. The case for tackling the threat cannot be so clearly separated from the measures used to tackle it. In *Ground Operation*, the collateral damage to innocents should be factored into the decision about which threat to pursue. It may be that, once we include this, it is preferable to tackle mass atrocities in South Sudan rather than to engage in a ground operation in Burkina Faso, given that the positive incentives would be non-coercive. Analogously, when we are considering where to go on holiday, we do not simply consider where we want to go and then decide the best way of getting there. We consider the difficulty of getting there alongside where we go. If we are travelling with young children, this may mean that, if based in Europe, we choose to go to the Algarve, given the short flying time, rather than the more idyllic Maldives. Thus, it is vital to consider the case for various measures to tackle different threats. This cannot be captured straightforwardly by adding an opportunity costs principle that is prior to necessity and proportionality. We can grasp cases such as *Ground Operation* only when we assess, *concurrently*, both forms of consideration – threat-specific considerations and measure-specific considerations. We need to know which threat should be addressed *alongside* which measures should be used in response to which threat.

How then should we understand opportunity costs in relation to Just War Theory? I suggest that we use the notion of ‘holistic necessity’. Holistic necessity requires the (concurrent) comparison of war both to other measures to address the threat in question *and to other measures to address other threats*. Holistic necessity therefore differs from necessity, as it is commonly understood in recent Just War Theory and the morality of self-defence, which we can call ‘discrete necessity’. Discrete necessity compares the measure in question only to other measures in response to *a single threat*, such as war to address aggression to economic sanctions to address aggression.^39^ This is akin to the principle of necessity *ad bellum* or, on some formulations, last resort. Accordingly, holistic necessity includes both the (1) threat-specific considerations and (2) measure-specific considerations, whereas discrete necessity considers only (2) measure-specific considerations.^40^

To summarise: the (largely brief) prevailing accounts of opportunity costs often focus on the threat-specific considerations. These do provide some reason to take opportunity costs seriously. However, they largely overlook the measure-specific considerations related to the tackling of other threats. This seems to make it even harder for war to be permissible. This is because not only is there a lot more going for the alternatives to war to address the threat in question, there is also a lot more going for other measures to address *other threats*. This is what the notion of holistic necessity captures.

This brings us to the crux: in addition to the four reasons in favour of pacifism noted in the previous section, there is a fifth. That is, there are (1) instrumental and (2) deontological criticisms of war, (3) positive reasons in favour of the *other measures* to address the threat at hand (which is the typical focus of necessity or last resort), and (4) reasons to address *other* threats. In addition, there are (5) positive reasons in favour of the *other measures* to address *other threats*. This helps to explain further why the Opportunity Costs Objection might appear to take us to Opportunity Costs Pacifism.

## Responses

Let’s now consider replies to Opportunity Costs Pacifism. I will start with three replies that are largely unsuccessful, before considering a more plausible response in the next section.

### Response 1: Deterrence

The first response is that Opportunity Costs Pacifism is mistaken because there needs to be a large military presence in order to deter aggressors from launching an attack (when Opportunity Costs Pacifism is not internalised by all states). If there are no armies, or only weak ones, those who are currently in possession of weapons (or those who can obtain weapons), would be able to launch aggressive wars successfully when they choose. Moreover, the objection continues, without a robust, military response to aggression, aggression will appear to be condoned or tolerated, and this will encourage other states to engage in aggression in the future. Thus, Opportunity Costs Pacifism leaves one vulnerable to aggressors.^41^

In reply, there are other robust responses to aggression that deter and that do not involve engaging in military force. These include economic sanctions, criminal prosecutions, and diplomatic sanctions, such as naming and shaming and expulsion from international bodies. Such measures can impose significant costs on aggressors and make it clear that aggression is not tolerated or condoned. In addition, even without engaging in military force against aggressors, there can still be the *threat* of military force. It is important here to separate the issues of (1) the permissibility of *waging war* and (2) the justifiability of *military spending*. The Opportunity Costs Objection, and Opportunity Costs Pacifism in turn, are focused on the former. In regard to the latter, some military spending might still be justified on Opportunity Costs Pacifism, in order to provide a threat to deter internal and external aggressors.^42^ It is worth noting that, to sustain this threat, there need not necessarily be large standing armies. States might, for instance, have small, well-trained armies, with collective security arrangements with other states, or possess weapons of mass destruction. Thus, Opportunity Costs Pacifism does not necessarily require disarmament; it focuses on the resources used for (2) *wars*, rather than for (1) *maintaining the military more generally*, which may still be required. Notwithstanding, it seems likely that states spend far beyond that which is necessary to deter aggressors.^43^

### Response 2: Duties to Citizens

The second response to Opportunity Costs Pacifism holds that states are permitted – and indeed obliged – to wage wars even when they are suboptimal. This is (1) because of states’ fiduciary duties to promote their citizens’ interests and (2) because citizens are free to support suboptimal wars and states are duty-bound to represent their citizens’ opinions.

First, let’s consider the claim that states have very weighty fiduciary duties to promote their citizens’ interests. It follows, on this view, that wars of self-defence can be permissible (and indeed required), even if there are sizeable opportunity costs for noncitizens, given that the duty of the state is first and foremost to look after its own citizens. How plausible one finds this claim will depend, in part, on one’s views on the broader debate between cosmopolitans and statists about the strength of duties to fellow nationals vis-à-vis cosmopolitan duties.^44^ Although I cannot defend it at length here, my view is that there are weighty positive duties to noncitizens. On this view, this response to Opportunity Costs Pacifism fails since states are not permitted (or required) to give significant priority to their citizens by waging significantly suboptimal wars of self-defence. But let’s suppose that I am mistaken and that states’ positive obligations to those beyond their borders are minimal. I want to now argue that, *even then*, Opportunity Costs Pacifism seems plausible. This is for three reasons.

First, wars raise *domestic* opportunity costs, in addition to the international opportunity costs that I have focused on thus far. Many of the resources that states use to fight wars could be otherwise spent domestically. They could help to tackle domestic poverty and preventable deaths.^45^ For instance, the several trillion dollars spent on the wars in Iraq and Afghanistan could have been spent on providing adequate healthcare coverage in the U.S., which could have saved thousands of lives – and seemingly saved far more U.S. citizens than were at risk from the threats posed by Saddam Hussein’s Iraq and the Taliban regime in Afghanistan. For instance, a leading study finds that as many as 44,000 people die in the U.S. per year due to a lack of health insurance, which is far more than were perceived to be at serious risk on an even expansive notion of the threat posed by Iraq and the Taliban.^46^ Accordingly, even if there are very weighty fiduciary obligations, war appears likely to be highly suboptimal given potential domestic opportunity costs.

Second, as noted above, the Opportunity Costs Objection concerns not only positive duties to assist, but also negative duties not to harm, and war will almost always transgress negative duties not to harm. Most notably, it will typically *do* significant harm to (innocent) non-citizens. Although the positive duties of the state to citizens might outweigh *positive* duties to assist noncitizens, *negative* duties not to harm are widely viewed as very weighty, regardless of the nationality of the right-holder. At the individual level, strong positive associative duties do not seem to provide licence to significantly transgress negative duties. For instance, it seems that a father could not permissibly kill two other innocent children in order to protect his own child, despite the very weighty positive obligations of fatherhood.^47^ In a similar vein, it seems that a state could not permissibly harm several innocent civilians in another state in order to promote the basic interests of a few of its citizens. To see this, suppose that launching a bombing campaign against a terrorist threat abroad would decrease the risks to a few citizens, but would be likely to harm numerous civilians collaterally in the target state. It would seem problematic to launch the campaign, given its harming of noncitizens. Indeed, the leading accounts of proportionality by Adil Haque and Jeff McMahan reject the notion that national partiality justifies the deaths of a much greater number of foreign civilians.^48^ Part of the reason given by Haque and McMahan is that national partiality does not outweigh *doing* harm to foreign civilians by means of war, when there is the option of doing nothing.^49^ A similar logic applies to opportunity costs: fiduciary duties do not outweigh the wrongness of *doing* harm to more noncitizens by means of war, when there is the option of measures that would do less harm in tackling other threats.

Third, wars will also typically transgress negative duties not to harm the state’s *own citizens*; in short, wars *do* harm to citizens. Most clearly, wars may sometimes involve conscription. But even if based on an all-volunteer force, wars will often involve soldiers bearing significant harms, who have been misled into joining the armed forces by recruiters.^50^ Wars will also often harm the family and friends of those sent to war when those sent to war are injured or psychologically traumatised by war, as well as damage significantly the basic infrastructure of the state.

To overcome these three points, the benefits to citizens would have to be large enough to overcome the domestic opportunity costs and outweigh the wrongness of *doing* harm to both noncitizens and citizens. A war would, for instance, need to prevent the violation of basic rights of a much greater number of citizens than the number of noncitizens and citizens that it harms, and be the optimal way to promote citizens’ rights. Even if certain wars in the past *might* have met this threshold, it seems highly unlikely that currently, or in the near future, a war would do so. This is, in part, because it is doubtful whether states will face a significant enough threat from aggressors beyond their borders, which could require a major war of self-defence. Important here is that there has been a substantial decline in interstate aggression over the past 50 years.^51^ There are unlikely to be state aggressors waging large-scale offensives. As a result, it does not appear to be likely that states will face a sizable, existential threat to wage a war of self-defence.

Let’s now turn to the second response. Can the wishes of citizens mean that states can permissibly wage suboptimal wars of self-defence? To start with, it is worth noting that citizens often oppose war, particularly in the wake of the wars in Iraq and Afghanistan.^52^ This calls into question whether the demos is in fact likely to support foreign wars (to be sure, citizens are also often sceptical about using the resources elsewhere, such as for humanitarian assistance, preferring spending on domestic causes).^53^

More fundamentally, it seems doubtful that support from the demos *for* war should be viewed as a weighty factor when considering whether to go to war. To be sure, if the demos *opposes* war, this is a notable (if not necessarily insurmountable) constraint on waging war. This is because citizens will often have to bear the burdens of the war, such as increased taxation and casualties amongst friends and family in the armed forces.^54^ However, when the demos is in *favour* of war, this does not seem to be an important consideration. That is to say, citizen support is best seen as a *negative* constraint against launching a war rather than a *positive* reason in favour of launching a war. This is because wars, again, tend to *do* harm. The positive reasons in favour of respecting democratic wishes of the population do not seem to outweigh the stringent negative duties not to harm citizens and noncitizens. Indeed, at the domestic level, it seems uncontroversial that majority support from the demos does not permit a state to engage in rights violations of a minority. Similarly, at the international level, majority support for war does not seem to be sufficient to permit the state to engage in the transgression of the rights of those beyond the borders of the state (as well as of some of those within it).

It is worth considering one further point here. It might be thought that states are permitted to fight suboptimal wars if they so choose. The reasoning draws on the domestic analogy: just as individuals should be free to choose which good ends to pursue as long as they make a reasonable effort to tackle injustice, so should states be free to choose which good ends to pursue, as long as they make a reasonable effort to tackle injustice. We can now see why this analogy is mistaken. It is not simply because, as effective altruists argue, the first premise is wrong: individuals are not, in fact, free to choose which good ends they pursue – they should pursue optimal policies (when they can do so at no additional cost).^55^ It is also because, even if one denies effective altruism, it still seems clear that states are not free to choose which ends they pursue and how they do so. Most obviously, leaders of states cannot act on a whim. They are, at the very least, significantly constrained by their responsibilities to their citizens; they have responsibilities to consider the costs of a policy for citizens and to respect the wishes of the demos to an extent.^56^ Even if the costs to citizens are very low, or if a measure is supported by the demos, states are also constrained by their responsibilities not to harm noncitizens (as well as the positive duties towards noncitizens). These constraints mean that the leaders of state are not free to choose, on a whim, to wage a suboptimal war.^57^

### Response 3: Specific Aims of War

A third response to Opportunity Costs Pacifism concerns the aims of war. First, it might be thought that there are some values that *only war* can defend. These might be thought to include, for instance, deterring aggression, securing territory, or upholding political independence, and, in general, civil and political rights. One obvious problem with this response is that these values can sometimes be protected by other measures, such as economic sanctions, nonviolent action, or diplomatic action. Another version of the response holds that war *better defends* these values, whereas the other options are more suited to defend other values – especially socio-economic rights – such as tackling unnecessary deaths from global poverty. But this seems empirically dubious. Other measures can often be far more effective at defending these values, such as civil and political rights, than war is. Perhaps most clearly, war is a notoriously problematic measure to install democracy, which may be far better secured by supporting local movements non-violently.^58^ In addition, even when war will protect civil and political rights, it seems likely that the other options are typically likely to benefit (1) many more individuals (2) in greater ways in terms of socio-economic rights. That is, the number of individuals affected and the magnitude of the benefit to them are likely to be far higher. For instance, the aggregate benefits that accrue to individuals from tackling global poverty, such as access to healthcare, education, shelter, food, and medicine, would be likely to be greater than for war, in terms of the number of individuals benefitted and the size of benefit for each. War might, perhaps, provide aggregate benefits that accrue from protecting political independence or securing territory, but these seem likely to benefit fewer individuals (such as when defending the territory of a particular region) or be comparatively less significant (such as when upholding political independence).^59^

It might be replied here that Opportunity Costs Pacifism, as I have framed it, is premised upon a highly individualistic account of value. It is focused on the opportunity costs for individuals, for instance, of tackling communicable and noncommunicable diseases. In doing so, it overlooks *collective* values, such as self-determination. War might be thought justified in protecting such collective values. However, the most plausible accounts of collective values ultimately hold that such values are reducible to their value to individuals.^60^ When this is the case, it seems clear that they can be outweighed by the greater value to individuals derived from tackling other threats. For instance, the individual value in self-determination is likely to be outweighed by the individual value in being free from unnecessary disease. Even if one does not accept this, there is, again, a more straightforward response. Many of the collective values defended by war are under greater threat from disease and other forms of violence, such as domestic violence against women. For instance, the huge burden of disease can make it extremely difficult for a community to be collectively self-determining, at least in a meaningful sense. It will not enjoy the freedom from want necessary for the community to be able to self-determine.

There is also a different argument about the ends of war. This comes from Oberman, who argues that the opportunity costs of war and poverty “take us a step towards pacifism, making war much harder to justify. They do not, however, take us all the way”.^61^ This is because Oberman sees necessity/last resort as comparing effects limited by the potential ‘just ends of war’. When the opportunity costs concern what could be the just ends of war, such as tackling a humanitarian disaster, then war can be unnecessary. However, for Oberman, war can still be necessary when it is the best option to achieve the ‘just ends of war’, such as defence against an invasion, and the opportunity costs do not concern the just ends of war, perhaps such as health or education.^62^

However, it is unclear why exactly for Oberman opportunity costs do not take us to pacifism – or, at least, tip the balance in favour of pacifism when combined with the other reasons in favour of it.^63^ First, it seems to confuse matters to refer to the ‘just ends of war’ in this context. As argued above, we need a broader account of necessity – holistic necessity – that is not limited to considering whether the measure should be launched to tackle the *threat in question*; it should also include considering whether *other* threats should be tackled. Crucially, these other threats should not be limited to only the ‘just ends of war’. Rather, they should include threats that are not widely thought to be a just end of war, such as health and education. To see this, suppose that a state faces the choice whether to (1) launch a war to tackle a terrorist threat or (2) to use these resources to improve education in less developed countries, which will have the effect of educating thousands of underprivileged children. It still seems that the state should take seriously the fact that war would use resources that would preclude education. The comparison made should therefore not be limited to the ‘just ends of war’.

Suppose, though, that one still wants to hold that the just ends of war provide a limit on the threats that can be tackled, as Oberman suggests. If we do so, it seems plausible that there should be a very expansive list of ends. In principle, a war could pursue many goals, from tackling poverty to saving the lives of non-human species.^64^ What’s more, if we adopt a broad account of the just ends of war, there are also many just ends that can be pursued by other measures. For instance, tackling poverty might be a just end of war. If so, it is permissible to compare war to measures that tackle poverty, even if the war in question has a different aim. Indeed, Oberman adopts a very broad account of the just ends of war, including self-defence, the prevention of mass atrocities, achieving basic well-being, and potentially law enforcement and punishment. If so many other ends can be justifiably pursued, it seems likely that war will often be unnecessary, and so the objection to Opportunity Costs Pacifism falters.^65^

## War permissible nonideally

Having considered three responses to Opportunity Costs Pacifism that are unpersuasive, I will now present a fourth. This does show that it is sometimes permissible for states to go to war. However, it still accepts that opportunity costs take us to pacifism. How can this be coherent?

Unfortunately, we can expect parliaments and populations often *not* to be motivated by tackling many of the threats facing those beyond their borders. They will often ignore opportunity costs that arise when considering war. They will focus on tackling vivid, spectacular threats, such as those posed by terrorism, with little regard for the cost. Militarised responses are deeply ingrained, as has been all too clear with the Global War on Terror. As such, populations and parliaments may not support action, for instance, to tackle infectious disease over military action to tackle terrorist threats or even mass atrocities. It seems highly unlikely, for example, that the U.S. public and Congress more generally would have supported anything even close to the $5.9 trillion spent on the Global War on Terror and wars in Iraq and Afghanistan being used instead to eradicate global poverty and preventable disease in other states.

Governments and leaders may sometimes find these to be serious, robust constraints. They will lack the domestic support necessary to prioritise threats in the most ethical way and attempts may be blocked. The only option that will not be blocked will be a military response. What this means is that the real choice facing governments will sometimes not be between, for instance, tackling mass atrocities or infectious disease, but rather between tackling mass atrocities through war and doing nothing. Another way of putting this is that questions of opportunity costs sometimes do not arise because they are not within the feasible option set of decision-makers.^66^

This leaves the door open for war to be permissible non-ideally. The decision to go to war might still be permissible, given the good that it will bring about, compared to the other feasible option (of doing nothing). For instance, in the case of Libya, it seems highly likely that the U.S. and U.K. publics and legislatures would have not supported the resources used to intervene being used instead to increase significantly the aid budget. The choice, it seems, was to act or not, and, as such, the decision to intervene might still have been permissible. In this sense, holistic necessity is not, in fact, a necessary condition of *jus ad bellum*. Wars can be permissible even though suboptimal.

In a somewhat similar vein, Tadros argues that “[i]t is often easier to motivate people and states to support and engage in humanitarian wars to prevent genocide, or other seriously wrongful acts, than it is to motivate people to provide resources for other kinds of humanitarian aid”.^67^ Thus, he argues, we should not deter or condemn humanitarian intervention, “even though it is wrong to engage in” it since doing so will not get states to do what they should by spending resources on other humanitarian projects.

My point goes further: sometimes governments *do not act wrongly* in such cases since there is not the feasible option of using the resources otherwise. The reason to condemn them is not for instrumental reasons. Rather, it is because they simply have not done anything that merits condemnation. They have acted rightly in the circumstances. This is consistent with holding that the public and others who maintain the constraints on decision-makers should be condemned.

## Broader implications

This has major implications for Just War Theory and for pacifism. A *highly* non-ideal morality of war is the only place where there can really be the domain of Just War Theory. It is only at this extremely non-ideal level that we can talk of morally permissible wars in practice. At this level, there will be non-compliance not simply by the aggressors that provide the just cause, but also severe non-compliance by many of those in the responding state, which places severe restrictions on policymakers in this state. Above this level, Opportunity Costs Pacifism seems plausible, given the opportunity costs involved with war.^68^

To elaborate, non-ideal theory (in the Rawlsian sense) concerns circumstances where there is non-compliance with the ideal moral principles and the existence of favourable circumstances in order to realise the ideal (such as historical, economic, and social conditions). Accounts of Just War Theory are all forms of non-ideal theory given that they take into account non-compliance with the ideal moral principles by aggressors, but vary in how much they reflect non-compliance and unfavourable circumstances, with different degrees of ‘ideality’. Roughly, traditionalists often offer more non-ideal accounts; revisionists often offer more idealised accounts.^69^ My point here is that the potential for wars to be permissible will concern only cases where there are *hugely* unfavourable circumstances. This is where decision-makers cannot tackle threats in a more peaceful manner given domestic constraints. There is non-compliance by those upholding the constraints, such as by parliamentarians and the public (i.e. non-compliance with the requirement to tackle the most serious threats in the most peaceful manner). Just War Theory is plausible only when the decision-makers’ option set is circumscribed in this way. This is far more non-ideal than prevailing accounts of Just War Theory, both revisionist and traditionalist. Even the more practical and non-ideal focused accounts do not reflect this sort of non-compliance and unfavourable circumstance.

This is also important for pacifism – and not simply for Opportunity Costs Pacifism. The worry that leaders will not be able to choose the best response also applies to the choice of means to tackle a particular threat, that is, discrete necessity, which, as noted above, is more commonly understood as last resort or necessity *ad bellum*. Sometimes certain measures, such as offering positive incentives, might be far more likely to be justified than military action, but face significant domestic constraints, such as being unpopular domestically.^70^ Other measures, such as EU or UN economic sanctions or an arms embargo, might fail to receive the international backing necessary to be launched. As such, in addition to the fourth and fifth reasons in favour of pacifism noted above, the third reason – that there are alternatives that can better tackle the threat in question – also fails to apply in hugely unfavourable circumstances. The choice, in effect, is between doing nothing and launching military action in response to the threat in question. The plausibility of pacifism in large part depends on other threats or other measures being adopted. If they are unlikely to be so, war can still be permissible sometimes. Pacifism is far less appealing when the likely alternative is passivism.

## Conclusion

To conclude, I have argued that the opportunity costs of war appear to lead to pacifism, specifically, Opportunity Costs Pacifism. At the ideal level, Opportunity Costs Pacifism seems plausible. Wars are unlikely to be permissible, once the Opportunity Costs Objection is added to the other arguments against war. Notwithstanding, wars can still be permissible in highly nonideal circumstances, when the choices facing decision-makers are significantly constrained. The upshot is that it is only at the highly nonideal level that war can be justified and we can speak of just wars according to Just War Theory.

## Footnotes

1 Neta Crawford, ‘United States Budgetary Costs of the Post-9/11 Wars Through FY2019: $5.9 Trillion Spent and Obligated’, Costs of War Project, November 2018.

2 See, for instance, Iain Atack, *Nonviolence in Political Theory* (Edinburgh: Edinburgh University Press, 2012); Duane L. Cady, *From Warism to Pacifism: A Moral Continuum*, Second Edition (Philadelphia: Temple University Press, 2010); Andrew Fiala, *Practical Pacifism* (New York: Algora, 2004); Robert L. Holmes, *On War and Morality* (Princeton, N.J.: Princeton University Press, 1989); Robert L. Holmes, *Pacifism: A Philosophy of Nonviolence* (London: Bloomsbury, 2016); Larry May, *Contingent Pacifism: Revisiting Just War Theory* (Cambridge: Cambridge University Press, 2015). As far as I am aware, there are no accounts of pacifism that explore the issue of opportunity costs in detail. To be sure, opportunity costs are sometimes noted by pacifists as a reason to oppose war, but this is often fleeting.

3 See, for instance, Thomas Hurka, ‘Proportionality and Necessity’, in Larry May (ed.), *War: Essays in Political Philosophy*, (Cambridge: Cambridge University Press, 2008), pp. 127–144, at p. 134.

4 Jeff McMahan, ‘Proportionate Defense’, *Journal of Transnational Law and Policy* 21 (2013–2014): pp. 1–36, at 2–6; Kieran Oberman, ‘War and Poverty’, *Philosophical Studies* 176 (2019): pp. 197–217; Victor Tadros, *The Ends of Harm: The Moral Foundations of Criminal Law* (Oxford: Oxford University Press, 2011), pp. 338–345; Victor Tadros, ‘Unjust Wars Worth Fighting For’, *Journal of Practical Ethics* 4 (2016): pp. 52–78, at pp. 72–76. Opportunity costs are also briefly noted by Robin Dunford and Michael Neu, ‘The Responsibility to Protect in a World of Already Existing Intervention’, *European Journal of International Relations* 25 (2019): pp. 1080–1102, at pp. 1087–1089; Christopher Finlay, *Is Just War Possible?* (Cambridge: Polity Press, 2019), pp. 62–63; Uwe Steinhoff, ‘Is There a Duty to Militarily Intervene to Stop a Genocide?’, in Christian Neuhäuser and Christoph Schuck (eds.), *Military Interventions: Considerations from Philosophy and Political Science* (Baden-Baden: Nomos, 2017), pp. 59–80; and Benjamin A. Valentino, ‘The True Costs of Humanitarian Intervention: The Hard Truth about a Noble Notion’, *Foreign Affairs*, November/December, 2011: pp. 60–73.

5 This follows the influential and widely adopted definition of war given by the Correlates of War project. See Kristian Gleditsch, ‘A Revised List of Wars Between and Within Independent States, 1816-2002)’, *International Interactions* 30 (2004): pp. 231–262.

6 On these issues, see the seminal treatments by Christopher Finlay, *Terrorism and the Right to Resist: A Theory of Just Revolutionary War* (Cambridge: Cambridge University Press, 2015) and Michael Gross, *The Ethics of Insurgency: A Critical Guide to Just Guerrilla Warfare* (Cambridge: Cambridge University Press, 2015).

7 World Health Organization (WHO), *Global Health Estimates 2016 Summary Tables: Causes of Death, 2000–2016).* Available at <https://www.who.int/healthinfo/global_burden_disease/GHE2016_Deaths_Global_2000_2016.xls?ua=1>. Also see Causes of Death Collaborators, ‘Global, Regional, and National Age-sex-specific Mortality for 282 Causes of Death in 195 Countries and Territories, 1980–2017: A Systematic Analysis for the Global Burden of Disease Study 2017’, *The Lancet*, 392 (2018): pp. 1736–1788.

8 Valentino, ‘The True Costs of Humanitarian Intervention’ p. 69. The COVID-19 pandemic (which, at the time of writing, is developing) is likely to affect these figures, rendering violence an even smaller proportion of the overall annual death toll.

9 Toby Ord, ‘The Moral Imperative Toward Cost-effectiveness in Global Health’, Center for Global Development, March 2013, p. 4; Jenifer Ehreth, ‘The Global Value of Vaccination’, *Vaccine* 21 (2003): pp. 596–600.

10 This is also a major line of argument from effective altruists. See William MacAskill on ‘neglectedness’, *Doing Good Better: How Effective Altruism Can Help You Make a Difference* (London: Guardian Faber Publishing, 2015).

11 World Health Organization, *Antibacterial Agents in Clinical Development: An Analysis of the Antibacterial Clinical Development Pipeline, Including Tuberculosis*, September 2017. Available at <http://apps.who.int/iris/bitstream/10665/258965/1/WHO-EMP-IAU-2017.11-eng.pdf>.

12 Alex Braithwaite, Niheer Dasandi, and David Hudson, ‘Does Poverty Cause Conflict? Isolating the Causal Origins of the Conflict Trap’, *Conflict Management and Peace Science* 33 (2016): pp. 45–66.

13 WHO, *Global Health Estimates*.

14 Vikram Patel and Martin Prince, ‘Global Mental Health: A New Global Health Field Comes of Age’, *JAMA* 303 (2010): pp. 1976–1977. Moreover, mental, neurological and substance-use disorders constitute 13% of the global burden of disease (surpassing both cardiovascular disease and cancer), with depression as the third-leading contributor to the global disease burden. Pamela Y. Collins, Vikram Patel, Sarah S. Joestl, *et al.* ‘Grand Challenges in Global Mental Health’, *Nature* 475 (2011): pp. 27–30.

15 WHO, *Global Health Estimates.*

16 Samuel Scheffler, ‘Doing and Allowing’, *Ethics* 114 (2004): pp. 215–239; Fiona Woollard, ‘The Doctrine of Doing and Allowing I: Analysis of the Doing/Allowing Distinction’, *Philosophy Compass* 7 (2012): pp. 448–458; Fiona Woollard, ‘The Doctrine of Doing and Allowing II: The Moral Relevance of the Doing/Allowing Distinction’, *Philosophy Compass* 7 (2012): pp. 459–469.

17 See, for instance, David Miller, *National Responsibility and Global Justice* (Oxford: Oxford University Press, 2007); Thomas Nagel, ‘The Problem of Global Justice’, *Philosophy & Public Affairs* 33 (2005): pp. 113–147.

18 Ben Quinn, ‘UK Among Six Countries to Hit 0.7% UN Aid Spending Target’, *The Guardian*, 4 Jan 2017.

19 Fiala, *Practical Pacifism*; Andrew Fiala, ‘Practical Pacifism, *Jus in Bello*, and Citizen Responsibility: The Case of Iraq’, *Ethical Perspectives: Journal of the European Ethics Network* 13 (2006): pp. 673–697. In a similar vein, Andrew Alexandra presents his approach as ‘political pacifism’ that rejects the ‘institution of war’ but appears to view some wars as still permissible. Andrew Alexandra, ‘Liability, War, and Peace’, *Philosophical Forum* 46 (2015): pp. 41–53, at p. 42; Andrew Alexandra, ‘Political Pacifism’, *Social Theory and Practice* 29 (2003): pp. 589–606. See, further, James Pattison, *The Alternatives to War: From Sanctions to Nonviolence* (Oxford: Oxford University Press, 2018), pp. 216–220; Cheyney Ryan, ‘Pacifism(s)’, *Philosophical Forum* 46 (2015): pp. 17–39; Cheyney Ryan, ‘Pacifism’, in Seth Lazar and Helen Frowe (eds.), *The Oxford Handbook of Ethics of War* (Oxford: Oxford University Press, 2018), pp. 277–293.

20 John Rawls, *A Theory of Justice*, Revised Edition (Oxford: Oxford University Press, 1999), p. 335. For a more detailed exploration of contingent pacifism, see Saba Bazargan, ‘Varieties of Contingent Pacifism in War’, in Helen Frowe and Gerard Lang (eds), *How We Fight* (Oxford: Oxford University Press, 2014), pp. 1–17 and May, *Contingent Pacifism*.

21 There are other arguments presented by pacifists; I focus on those that seem most telling.

22 Laurie Calhoun, ‘How Violence Breeds Violence: Some Utilitarian Considerations’, *Politics* 22 (2002): pp. 95–108.

23 Holmes, *On War and Morality*, pp. 203–211. This critique is also sometimes framed in terms of the wrongfulness of *intentionally* harming the innocent. Although I cannot pursue this here, it seems to me that the wrong at stake is in fact largely about *doing* harm to innocents, since the harms are often only collateral and, even when intended, may still be motivated by good reasons. Not much turns on this, though, for this article.

24 Cécile Fabre, *Economic Statecraft: Human Rights, Sanctions, and Conditionality* (Cambridge, Mass.: Harvard University Press, 2018); Pattison, *The Alternatives to War*.

25 Bazargan, ‘Varieties of Contingent Pacifism in War’; Jeff McMahan, ‘Pacifism and Moral Theory’, *Diametros* 23 (2010): pp. 3–20.

26 Tadros, ‘Unjust Wars Worth Fighting For’, pp. 73–74.

27 Oberman, ‘War and Poverty’, p. 197.

28 Peter Singer, ‘Bystanders to Poverty’, in N. Ann Davis, Richard Keshen, and Jeff McMahan (eds.), *Ethics and Humanity: Themes from the Philosophy of Jonathan Glover* (Oxford: Oxford University Press, 2010), pp. 185–201.

29 Singer, ‘Bystanders to Poverty’, p. 197.

30 McMahan, ‘Proportionate Defense’, pp. 2–6.

31 Dan W. Brock and Daniel Wikler, ‘Ethical Issues in Resource Allocation, Research, and New Product Development’, in Dean T. Jamison, Joel G. Breman, Anthony R. Measham, George Alleyne, Mariam Claeson, David B. Evans, Prabhat Jha, Anne Mills, and Philip Musgrove (eds.), *Disease Control Priorities in Developing Countries*, Second Edition (Washington, D.C.: The International Bank for Reconstruction and Development/The World Bank; New York: Oxford University Press, 2006), pp. 259–270; John Harris, ‘Deciding Between Patients’, in Helga Kuhse and Peter Singer (eds.), *A Companion to Bioethics*, Second Edition (London: Blackwell, 2017), pp. 335–350; Govind Persad, Alan Wertheimer, and Ezekiel J. Emanuel, ‘Principles for Allocation of Scarce Medical Interventions’, *The Lancet* 373 (2009): pp. 423–431.

32 These considerations are found widely in accounts of assessments of the ethics of war and of non-military measures, such as sanctions and diplomacy. See, for instance, Cécile Fabre, *Cosmopolitan War* (Oxford: Oxford University Press, 2012); Fabre, *Economic Statecraft*, and Pattison, *The Alternatives to War*.

33 I focus on health interventions in general here, rather than responses to global pandemics. As the global response to COVID-19 demonstrates, health interventions can sometimes be very coercive.

34 Tadros (2011: 338–345) sees them as part of proportionality; McMahan (2013–2014: 2–6) defends the need for a new principle; Oberman, ‘War and Poverty’, argues that they are part of both necessity and proportionality.

35 See, for instance, McMahan, ‘Proportionate Defense’, p. 3.

36 Seth Lazar, ‘War’, in Edward Zalta (ed.), *The Stanford Encyclopedia of Philosophy*, Spring 2017 Edition. Available at <https://plato.stanford.edu/archives/spr2017/entries/war/>. Also see Seth Lazar, ‘Necessity in Self-Defense and War’, *Philosophy & Public Affairs* 40 (2012): pp. 3–44.

37 McMahan, ‘Proportionate Defense’, pp. 2–6.

38 To be sure, an additional opportunity costs principle, such as that proposed by McMahan, could run concurrently (i.e. alongside necessity and proportionality). (McMahan does not delineate how he sees his principle working). I focus on an opportunity costs principle that is used prior to necessity.

39 This is similar to the distinction drawn by Oberman between the ‘standard’ and ‘revisionary’ view of necessity. Kieran Oberman, ‘Killing and Rescuing: The Case for Revising Necessity’, *Philosophical Review*, forthcoming.

40 Holistic necessity might be thought to extend the concept of necessity too far. This is if necessity is seen as concerning whether the costs of a measure are necessary, so, for instance, a measure (such as war) will meet the necessity condition if it is the least harmful potential measure. Although I cannot consider this point further here, this strikes me as a too restrictive understanding of necessity. What necessity ultimately captures is the importance of comparing war to the alternatives. The costs of the various measures are an important issue in this assessment, but there are other considerations – i.e. other measure-specific considerations – including whether the other measures will save more lives, how the measures will fare according to the Doctrine of Doing and Allowing, and the fairness of the distribution of any costs and benefits. In my defence of holistic necessity, I have argued that this comparative assessment should reflect both measure-specific and threat-specific considerations. (I frame this in terms of necessity because this is the principle that is usually used to cover such comparative assessments.) Notwithstanding, if one wants to adopt a narrow view of necessity (e.g. limiting it only to costs), then another term rather than holistic necessity may be preferable, such as ‘holistic comparative assessment’. Not much of substance turns on this.

41 Finlay, *Is Just War Possible?*, pp. 62–63.

42 For the deterrent threat to work, states might be required to bluff, that is, give the impression of being willing to go to war.

43 It is also possible that agreements made between states might provide further reason in favour of war, such as when states make collective security arrangements. However, it seems unlikely that such agreements undermine the case for Opportunity Costs Pacifism, although space precludes considering this fully here. This is because the importance of upholding such agreements seems likely to be outweighed by the opportunity costs of war. That is, it would seem preferable for a state to break an agreement and save many more lives by avoiding war, than to uphold it and launch a severely suboptimal war.

44 See, for instance, Simon Caney, *Justice Beyond Borders: A Global Political Theory* (Oxford: Oxford University Press, 2005); Miller, *National Responsibility and Global Justice*; Nagel, ‘The Problem of Global Justice’; Thomas Pogge, *World Poverty and Human Rights*, Second Edition (Cambridge: Polity Press, 2008).

45 Oberman, ‘War and Poverty’, p. 203.

46 Andrew P. Wilper, Steffie Woolhandler, Karen E. Lasser, Danny McCormick, David H. Bor, and David U. Himmelstein, ‘Health Insurance and Mortality in US Adults’, *American Journal of Public Health* 99 (2009): pp. 2289–2295.

47 Alec D. Walen, *The Mechanics of Claims and Permissible Killing in War* (New York: Oxford University Press, 2019), pp. 78–79.

48 Adil Ahmad Haque, *Law and Morality at War* (Oxford: Oxford University Press, 2017); Jeff McMahan, ‘The Just Distribution of Harm between Combatants and Noncombatants’, *Philosophy & Public Affairs* 38 (2010): pp. 342–379.

49 Haque, *Law and Morality at War*, pp. 13–14; McMahan, ‘The Just Distribution of Harm’, p. 377.

50 For instance, the US Army has allegedly misled student recruits and to have targeted children in a manner akin to ‘predatory grooming’. Amy Hagopian and Kathy Barker, ‘Should We End Military Recruiting in High Schools as a Matter of Child Protection and Public Health?’ *American Journal of Public Health* 101 (2011): pp. 19–23; Lawrence Korb and Sean Duggan, ‘An All-Volunteer Army? Recruitment and its Problems’, *Political Science & Politics* 40 (2007): pp. 467–471.

51 For an overview of the literature on the decline of war, see Nils Petter Gleditsch, ‘The Decline of War – The Main Issues’, *International Studies Review* 15 (2013): pp. 397–399. Recall here that I focus on international conflicts; states might still face internal threats, such as those posed by rebel groups (levels of intrastate conflict have not experienced such a degree of decline).

52 For instance, although public opinion on warfare can vary, a recent YouGov survey suggests that the majority of populations in Western states, including the U.S., France, and U.K., oppose foreign interventions. YouGov, ‘Eurotrack: Corbyn’s Policies Popular in Europe and UK’, 2019. Available at <https://yougov.co.uk/topics/politics/articles-reports/2019/01/09/eurotrack-corbyns-policies-popular-europe-and-uk>. The public support for war, even if it is successful, decreases as they become aware of diplomatic alternatives. Aaron M. Hoffman, Christopher R. Agnew, Laura E. VanderDrift, and Robert Kulzick, ‘Norms, Diplomatic Alternatives, and the Social Psychology of War Support’, *Journal of Conflict Resolution* 59 (2013): pp. 3–28.

53 For instance, the U.S. public tends to be sceptical of aid, with the majority believing that it gives too much. Reuben Hurst, Taylor Tidwell, and Darren Hawkins, ‘Down the Rathole? Public Support for US Foreign Aid’, *International Studies Quarterly* 61 (2017): pp. 442–454.

54 See, further, James Pattison, ‘Representativeness and Humanitarian Intervention’, *Journal of Social Philosophy* 38 (2017): pp. 569–587. Of course, citizens may also oppose launching other options, such as economic sanctions because they will have to bear very significant burdens. This might count against launching such measures, although not necessarily *in favour* of war (doing nothing may be the preferred option).

55 MacAskill, *Doing Good Better*; Theron Pummer, ‘Whether and Where to Give’, *Philosophy & Public Affairs* 44 (2016): pp. 77–95; Peter Singer, *The Most Good You Can Do: How Effective Altruism is Changing Ideas About Living Ethically* (New Haven: Yale University Press, 2015). To be sure, effective altruism could still permit space for individuals to pursue their own projects. The key point is that, when individuals are pursuing good ends, they should pursue optimal ones (other things being equal). In other words, they should do the most good that they can do when they are doing good (other things being equal), but still might have space not to have to do good all the time.

56 On this issue, see further Kieran Oberman, ‘The Myth of Optional War: Why States are Required to Wage the Wars They Are Permitted to Wage’, *Philosophy & Public Affairs* 43 (2015): pp. 255–286. Oberman argues that there cannot be supererogatory wars.

57 To be sure, a war could, conceivably, not raise significant domestic opportunity costs and not transgress significantly negative duties not to harm citizens and noncitizens. However, such cases are likely to be rare, given that wars do tend to transgress significantly negative duties and raise domestic opportunity cases. Moreover, in such cases, the positive duties that I have bracketed would be relevant and would be likely to tip the balance in favour of Opportunity Cost Pacifism. That is, states would not be permitted (or required) to give significant priority to their citizens by waging hugely suboptimal wars of self-defence.

58 See Erica Chenoworth and Maria J. Stephan, *Why Civil Resistance Works: The Strategic Logic of Nonviolent Conflict* (New York: Columbia University Press, 2011). Chenoworth and Stephan find that nonviolent movements in general are far more likely to succeed than violent ones.

59 To be sure, I am not claiming that these values will *always* be better protected by these other methods. As such, there may be exceptions when war better promotes these values. But such exceptions seem likely to be rare and, even when they do apply, war is likely to do poorly according to other concerns, such as the import of avoiding *doing* harm or reducing overall harm. Again, it’s important to reiterate here that Opportunity Costs Pacifism is a form of contingent pacifism. Thus, even if there are *conceivable* exceptions where war not only better promotes these other values and where it does less harm and leads to fewer overall deaths, what matters is whether this is *currently feasible*. This seems highly unlikely.

60 For example, Moore, who offers the leading account of the values of territory and self-determination, powerfully defends the *individual* interest in these values. Margaret Moore, *A Political Theory of Territory* (Oxford: Oxford University Press, 2017). Moore also emphasises that these values are not absolute; they are constrained by individual human rights. Margaret Moore, ‘Reply to Critics’, *Critical Review of International Social and Political Philosophy* 21 (2018): pp. 806–817.

61 Oberman, ‘War and Poverty’, p. 215.

62 In addition to this argument about necessity, Oberman argues that opportunity costs can render war disproportionate. I argued above, though, that seeing opportunity costs as a matter of proportionality does not capture what is at stake, since proportionality compares to inaction (i.e. not launching the measure), rather than comparing to tackling other threats.

63 To be sure, Oberman generally focuses on tackling poverty as an opportunity cost. It is unclear whether he would hold that other opportunity costs, such as tackling climate change, would in fact take as all the way to pacifism.

64 Alasdair Cochrane and Steve Cooke, ‘Humane Intervention: The International Protection of Animal Rights’, *Journal of Global Ethics* 12 (2016): pp. 106–121; Fabre, *Cosmopolitan War*, pp. 97–128.

65 It might also be objected that it is not possible to compare fighting wars and tackling other threats because they are incommensurable. But this seems mistaken. After all, we compare war to other measures, such as economic sanctions, to address the threat at hand, under the principle of last resort and necessity *ad bellum*. More generally, it seems plausible that war can be compared to other measures by comparing (at least) the effect on basic interests. To that extent, there can be commensurability.

66 By feasible option-set, I mean what is within the scope of leaders’ official powers. These powers require them to have the approval of the legislature, for instance, for certain measures, and to be acting legally. Political unpopularity does not render an option unfeasible. Thus, that some non-military options are politically difficult would *not* be sufficient reason per se to go to war.

67 Tadros, ‘Unjust Wars Worth Fighting For’, p. 87.

68 To be sure, at the most idealised level of Just War Theory – a fully ‘deep’ morality of war – the existence of favourable circumstances (apart from the presence of an aggressor that provides just cause) would be fully assumed, including opportunity costs, and so war might still be justified. I focus here on more practically focussed accounts.

69 James Pattison, ‘The Case for the Nonideal Morality of War: Beyond Revisionism versus Traditionalism in Just War Theory’, *Political Theory* 46 (2018): pp. 242–268.

70 Graeme A. M. Davies and Robert Johns, ‘The Domestic Consequences of International Over-Cooperation: An Experimental Study of Microfoundations’, *Conflict Management and Peace Science* 33 (2016): pp. 343–360.

